# Nonvolatile ferroelectric field-effect transistors

**DOI:** 10.1038/s41467-020-16623-9

**Published:** 2020-06-04

**Authors:** Xiaojie Chai, Jun Jiang, Qinghua Zhang, Xu Hou, Fanqi Meng, Jie Wang, Lin Gu, David Wei Zhang, An Quan Jiang

**Affiliations:** 1 State Key Laboratory of ASIC & Systems, School of Microelectronics, Fudan University, 200433 Shanghai, China; 2 Beijing National Laboratory for Condensed Matter Physics, Institute of Physics, Chinese Academy of Sciences, 100190 Beijing, China; 3 Department of Engineering Mechanics, Zhejiang University, 310027 Hangzhou, China; 4 Key Laboratory of Soft Machines and Smart Devices of Zhejiang Province, 310027 Hangzhou, China; 5 University of Chinese Academy of Sciences, 100049 Beijing, China

**Keywords:** Electronic devices, Ferroelectrics and multiferroics, Electronics, photonics and device physics

## Abstract

Future data-intensive applications will have integrated circuit architectures combining energy-efficient transistors, high-density data storage and electro-optic sensing arrays in a single chip to perform in situ processing of captured data. The costly dense wire connections in 3D integrated circuits and in conventional packaging and chip-stacking solutions could affect data communication bandwidths, data storage densities, and optical transmission efficiency. Here we investigated all-ferroelectric nonvolatile LiNbO_3_ transistors to function through redirection of conducting domain walls between the drain, gate and source electrodes. The transistor operates as a single-pole, double-throw digital switch with complementary on/off source and gate currents controlled using either the gate or source voltages. The conceived device exhibits high wall current density and abrupt off-and-on state switching without subthreshold swing, enabling nonvolatile memory-and-sensor-in-logic and logic-in-memory-and-sensor capabilities with superior energy efficiency, ultrafast operation/communication speeds, and high logic/storage densities.

## Abstract (web summary)

There is growing interest in non-traditional materials for logic applications. Here, the authors demonstrate a domain device architecture based on ferroelectric LiNbO_3_ crystals with gate voltage controlled transistor without subthreshold swing and source voltage controlled nonvolatile transistor.

## Introduction

The frequent data shuttling between the physically separated processing and memory units in traditional digital computers incurs considerable penalties on the energy efficiency and data bandwidth, which is further intensified by the increasing disparity between the speed of the memory unit and the processor^1^. The latest generation of computing approaches requires memory devices to enable high-throughput, energy-efficient, and area-efficient information processing. Ferroelectric materials with spontaneous polarizations have piezoelectric, pyroelectric, and electro-optic properties and are widely used in nonvolatile memories, sensors, and electro-optic modulators that rely on heterogeneous integration of field-effect transistors (MOSFETs)^2–5^. Recently, there have been many observations of ferroelectric single crystals in which the domain walls are highly conductive^6–12^. Hall voltage measurements of ErMnO_3_ and YbMnO_3_ consistently indicated high mobilities of ~670 cm^2^ V^−1^ s^−1^ for active p-type carriers in tail-to-tail domain walls at room temperature^13,14^. These walls can be reversibly created, positioned and shaped using electric fields on a femtosecond time scale^15^, which is encouraging for future high-speed domain-wall nanoelectronics^16–18^. Although control over the injection of conducting domain walls between the source and drain electrodes has allowed landmark demonstration of three-terminal resistance memory devices^19,20^, they lack the superior abrupt on-and-off state switching capability of nonvolatile transistors. This functionality is pivotal for the performance of in situ data computing, storage, and sensing operations^21^.

Here we demonstrate such transistors using 5 mol% MgO-doped LiNbO_3_ (LN) monodomain single crystals that offer significantly enhanced diode-like wall currents^10,11^. LN single-crystal thin films can currently be produced by ionic slicing of surface layers off bulk crystals and chemical bonding of these layers to Si wafers, and have been widely applied in pyroelectric sensors, surface acoustic wave filters, and electro-optic modulators^3,4^. In this work, conducting drain–gate and drain–source conduits were complementarily created and erased using a controlled gate/source voltage through 180° reversal of the local domains. These junctionless transistors demonstrate low leakage, fast operating speeds (<5 ns), high wall currents (~110 μA μm^−1^), and abrupt off-and-on state switching without subthreshold swings (SS). In addition, these transistors offer uniform, programmable threshold voltages like those of the nanowire Ge/Si core/shell FET, with computing, memory, and addressing capabilities^22^.

## Results

### Device design and electrical measurement of LN FET

**Fig. 1:**
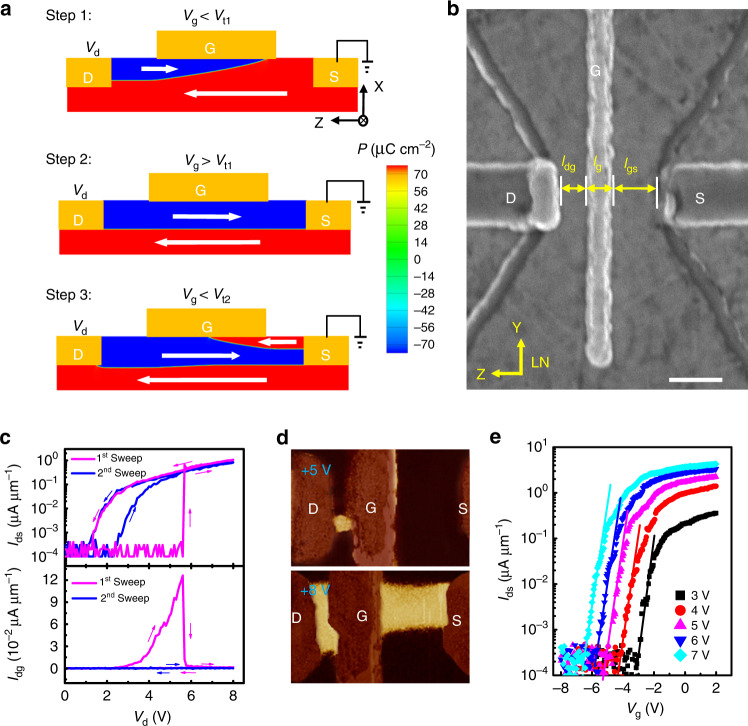
Working principles of domain-wall transistors. **a** Phase-field simulations of domain wall evolution for S2 as *V*_g_ varies from −8 V→ 0 V → −5 V in three steps with *V*_g_ < *V*_t1_, *V*_g_ > *V*_t1_, and *V*_g_ < *V*_t2_, where *V*_d_ = 8.5 V, *V*_s_ = 0, *V*_t1_ = −4.70 V, and *V*_t2_ = −4.72 V. Thick arrows indicate the domain orientations. **b** Planar SEM image of a typical three-terminal LN transistor (S9). The scalar bar: 200 nm. **c** *V*_d_ dependences of *I*_ds_ and *I*_dg_ when *V*_g_ = *V*_s_ = 0 during the first and second sweeps that confirm the nonvolatile D–G and D–S walls in S9 created through Steps 1 and 2. Thin arrows indicate the voltage sweeping directions. **d** In-plane PFM phase images of 180° domain reversal in the yellow-colored regions after application of various *V*_d_ when *V*_g_ = *V*_s_ = 0 V. **e** Semi-logarithmic *I*_ds_–*V*_g_ plots of field-effect currents across the unchanged D–S wall during backward *V*_g_ sweeping of S8 under application of various *V*_d_. Solid-line fits inferred an SS of 216 mV dec^−1^.

Figure 1a (Supplementary Fig. 1a–c) displays phase-field simulations of the switched domains (thick arrows) and conducting walls between the drain (D), gate (G), and source (S) electrodes for a cross-sectional three-terminal LN cell (S2) controlled via the gate voltage (*V*_g_) under application of sufficiently high drain voltage (*V*_d_) when the source voltage is grounded (*V*_s_ = 0). Because *V*_g_ is smaller than the forward threshold voltage (*V*_t1_), the electric field (*E*) between D and G is strong enough to reverse the D–G domain during head-to-head D–G wall formation, as shown in Step 1. The charged D–G wall in the thickness of 1.06 nm (inset in Supplementary Fig. 1a) is conducting and is partially screened by free electrons near G to compensate for the polarization charge (*P*) of 70 μC cm^−2^ to reduce the depolarization energy^23^. At this instant, the D–G current (*I*_dg_) is on but the D–S current (*I*_ds_) is off. When *V*_g_ increases above *V*_t1_, *E* strengthens between G and S, which allows the D–G domain to grow throughout the entire cell in forming a D–S wall to turn on *I*_ds_, as illustrated in Step 2. Simultaneously, the previous conduit between D and G is blocked (i.e., *I*_dg_ = 0). When *V*_g_ is reduced again below the backward threshold voltage *V*_t2_, the D–S domain contracts back into the Step 1 state to shut *I*_ds_ off via reversal of the local G–S domain in Step 3. This repetitive redirection of the conducting domain walls between D, G, and S appears similar to a single-pole, double-throw digital switch controlled by the gate voltage. Normally, *V*_t1_ ≠ *V*_t2_ because of hysteretic domain switching behavior that can be defined using the geometrical sizes of the samples (S1–13) listed in Table 1.

**Table 1:** Samples 1–13 with various geometrical sizes.

Sample	*l*_dg_ (nm)	*l*_g_ (nm)	*l*_gs_ (nm)	*h* (nm)
S1	70	200	70	55
S2	106	269	91	55
S3	127	242	130	55
S4	148	241	151	55
S5	175	397	192	55
S6	70	200	57	55
S7	128	250	139	55
S8	203	85	280	64
S9	112	57	189	64
S10	101	200	126	70
S11	117	50	163	55
S12	135	50	150	55
S13	106	63	111	65

Figure 1b shows planar scanning electron microscope (SEM) image of S9, where *l*_dg_ and *l*_gs_ are the distances between D and G and between G and S in widths of *w*, respectively, and the gate electrode width is *l*_g_. This mesa-like transistor was etched to a depth (*h*) of 64 nm with left and right side slopes (~80°) to contact D and S fully using Pt electrodes that were fabricated on an X-cut LN surface via electron-beam lithography (EBL) and dry etching processes (see the “Methods”). Figure 1c shows double *I*_ds_−*V*_d_ and *I*_dg_−*V*_d_ curves between 0 and 8 V when *V*_g_ = *V*_s_ = 0 V. During the first *V*_d_ sweep from 0 to 8 V, the initially off *I*_dg_ (*I*_ds_) begins to turn on (off) at 2.6 V before turning off (on) again at 5.7 V. Subsequently, the off (on) state is maintained until *V*_d_ drops to 0. The complementary on/off *I*_dg_ and *I*_ds_ follow the phase-field simulation results for wall redirection between G, D, and S in Fig. 1a (see Steps 1 and 2). During the next repeated sweep, *I*_dg_ (*I*_ds_) is always off (on above 2.2 V), indicating the nonvolatile natures of the D–G and D–S walls when created. Because the device was poled at an opposite voltage of −8 V, the on-and-off state switching above is reproduced in the first sweep. Subsequent in-plane piezoresponse force microscopy (PFM) phase imaging showed the remnants of the partially switched D–G domain after poling at +5 V (Step 1), which later extended throughout the D-S cell at +8 V (Step 2), as indicated by the yellow-colored regions in Fig. 1d.

When the D–S wall has been created, *I*_ds_ can be controlled using *V*_g_ through a major carrier accumulation/depletion mechanism within the unchanged D–S conduit without invoking of domain motion between D, G, and S, similar to a volatile MOSFET. Figure 1e shows semi-logarithmic *I*_ds_−*V*_g_ plots at different *V*_d_ values for S8 with much longer *l*_dg_ and *l*_gs_ to avoid D–S wall redirection during sweeping within the voltage range studied. Normally, the forward and backward on/off switching curves are hysteretic (Supplementary Fig. 2a) that can be minimized by reducing the D and S electrode contact heights to the LN mesa for S13 (Supplementary Fig. 2b). From the solid-line fittings shown in Fig. 1e, we consistently estimated SS values as high as 216 mV dec^−1^.

### *V*_*g*_-controlled transistor without subthreshold swing

**Fig. 2:**
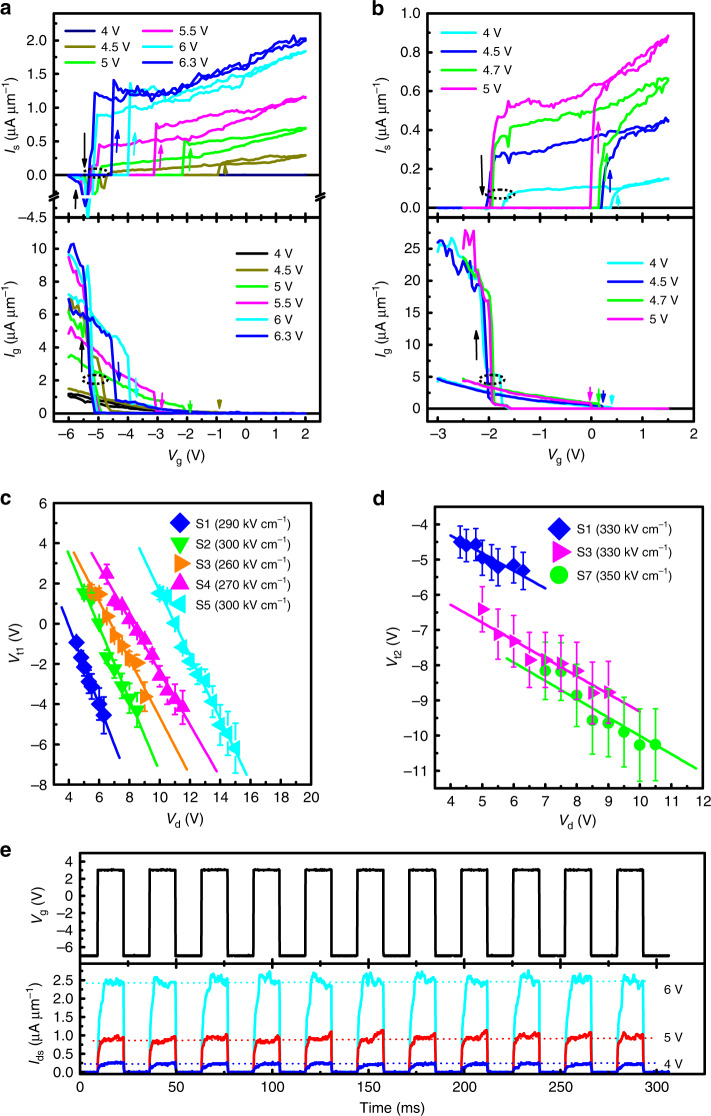
*V*_g_-controlled field-effect transistors. **a**, **b** *V*_g_ dependences of complementary *I*_s_ and *I*_g_ under application of various *V*_d_ with *V*_s_ = 0 for S1 and S6, respectively, where *I*_s_ turns on above *V*_t1_ (upward arrows) but turns off below *V*_t2_ (downward arrows); this contrasts with *I*_g_, which responds in the opposite manner. **c**, **d** *V*_d_ dependences of *V*_t1_ and *V*_t2_ for various samples fitted using solid lines based on Eqs. (1) and (2) with the extracted coercive fields shown in parentheses. **e** Time dependence of on/off *I*_ds_ transient for S4 when toggled using *V*_g_ pulses between −7 and 3 V in full duty at a frequency of 37 Hz under application of various *V*_d_ with *V*_s_ = 0 V. The error bars are defined as standard error of the mean.

Progressive downscaling of the operating voltages and device sizes of conventional MOSFETs in pursuit of multigenerational computing with superior energy efficiency and high integration density has reached the Boltzmann limit at SS no less than 60 mV dec^−1^ at room temperature^24^. This could be avoided in the DW transistors. To minimize the SS, *l*_dg_, and *l*_gs_ in S1–S5 were shortened so that the repetitive G–S domain switching would occur in Step 3 to turn off/on the complementary *I*_g_ and *I*_s_ by controlling either *V*_s_ or *V*_g_, where *I*_s_ = *I*_ds_ + *I*_gs_ and *I*_g_ = *I*_dg_ + *I*_sg_. For subscript description, *I*_sg_, for example, indicates the current flowing from S to G, where *I*_sg_ = −*I*_gs_. Figure 2a shows *V*_g_-controlled complementary *I*_s_ and *I*_g_ characteristics for S1 at various applied *V*_d_ when *V*_s_ = 0. During forward *V*_g_ sweeping from −6 to 2 V, *I*_s_ switches abruptly from off to an on-state above *V*_t1_ that varies from −0.93 to −4.54 V as *V*_d_ increases from 4 to 6.3 V; in contrast, *I*_g_ switches in the opposite manner. During subsequent backward sweeping of *V*_g_ from 2 to −6 V, *I*_s_ (*I*_g_) turns off (on) below *V*_t2_ (−4.30 to −5.32 V). Simultaneously, several overshoots of negative *I*_s_ peaks were observed near *V*_t2_ (see upper panel of Fig. 2a), indicating the temporary current flow occurring from S to G during wall redirection (from Step 2 to Step 1) through an intermediate Step 3 (Fig. 1a). When *l*_gs_ was shortened from 70 nm in S1 to 57 nm in S6, *V*_t2_ increased up to −1.95 V and the *I*_sg_ overshoots were too short to be detected (Fig. 2b), thus indicating the adjustable hysteresis of both *V*_t1_ and *V*_t2_. This abrupt on-and-off state switching advances the collective domain switching behavior that breaks through the SS limit of conventional MOSFETs. Figure 2c, d show typical *V*_t1_−*V*_d_ and *V*_t2_−*V*_d_ plots for the samples listed in Table 1. The two types of plots reflect the domain hysteretic switching behavior that can be adjusted by changing either the geometrical sizes (*l*_dg_, *l*_gs_, *l*_g_) or *V*_d_.

**Fig. 3:**
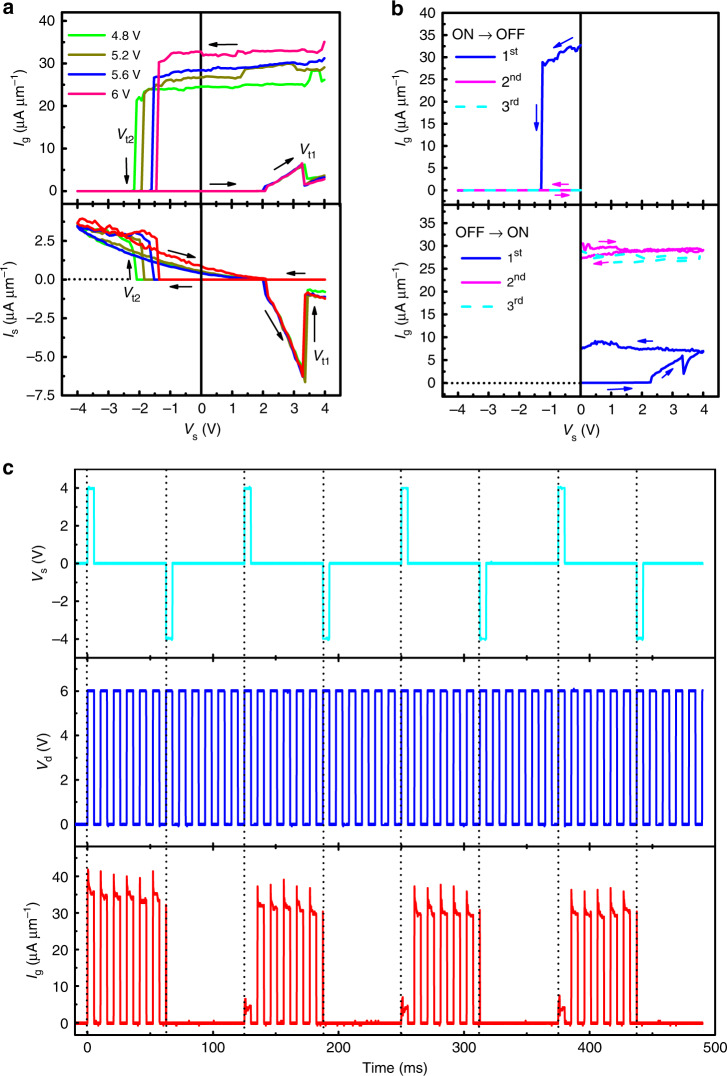
*V*_s_-controlled field-effect transistors. **a** *V*_s_ dependences of complementary *I*_g_ and *I*_s_ for S10 under application of various *V*_d_ with *V*_g_ = 0, where *I*_g_ turns off below *V*_t2_ but turns on above *V*_t1_; this contrasts with *I*_s_, which responds in the opposite manner. Arrows indicate the voltage sweeping directions. **b** On-to-off and off-to-on *I*_g_ toggled using the first sweep of *V*_s_, which varies either from 0 → −4 V → 0 (upper panel) or from 0 → 4 V → 0 (lower panel) when *V*_d_ = 6 V and *V*_g_ = 0. Each transition is nonvolatile, as confirmed by the next two repeated cycles. **c** Nonvolatile on/off *I*_g_ when intermittently accessed at a repeat frequency of 96 Hz with *V*_d_ = 6 V and *V*_g_ = 0 V, as toggled using *V*_s_ pulses between −4 and 4 V in 8.3% duty at a repeat frequency of 8.2 Hz.

Normally, domain switching initiates from reverse domain nucleation at the interface near D or S above a coercive field of *E*_c2_. When the domain exceeds a critical size in Step 1, it can penetrate throughout the entire cell in Step 2 under a much smaller driving field^23^. If *l*_g_ >> *h*, we approximately obtainwhere *E*_c1_ is the average coercive field for D–S domain growth (0 < *E*_c1_ < *E*_c2_). The solid lines in Fig. 3c, d are the best fits to the data according to Eqs (1) and (2), from which we found that *E*_c1_ = 260–300 kV cm^−1^ and *E*_c2_ = 330–350 kV cm^−1^, respectively.

The semi-logarithmic *I*_ds_−*V*_g_ plots for S2 when *V*_d_ = 8.5 V show the large on/off ratio (~10^4^) and abruptness (SS = 0) of the on-to-off and off-to-on state switching over 100 cycles (Supplementary Fig. 3a). However, *V*_t1_ and *V*_t2_ show broad dispersions (~0.8 V) over the cycles. These dispersions are believed to be correlated with etching damage to the D and S electrode contacts to an LN cell during device fabrication that also sets the upper limit for large current flows. To mitigate this damage, thick electroplated Ni electrodes were fabricated above Pt (Supplementary Fig. 3b). Subsequently, double *I*_ds_−*V*_d_ measurements showed a wall current as high as ~110 μA μm^−1^ at 656 kV cm^−1^ (Supplementary Fig. 3c), and after the initial three *I*_ds_−*V*_d_ sweeps, the coercive field levelled off at 284 kV cm^−1^ with negligible dispersion over next 120 cycles (Supplementary Fig. 3d). Unfortunately, this Ni electroplating technique cannot define fine sub-100 nm nanodevice patterns using academic facilities. Figure 2e shows successful on-and-off switching of *I*_ds_ transients for S4 when toggled using *V*_g_ pulses between −7 and 3 V at a frequency of 37 Hz under various applied *V*_d_.

### *V*_*s*_-controlled nonvolatile transistor

**Fig. 4:**
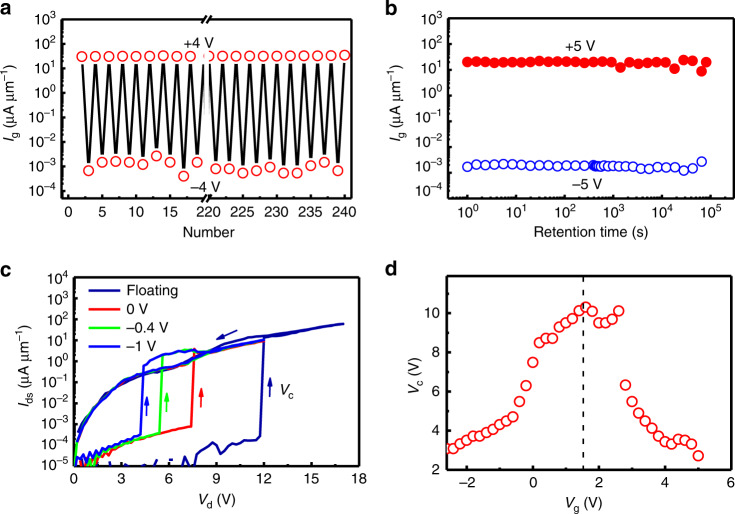
Reliability testing. **a** Switching number dependence of nonvolatile on/off *I*_g_ currents with *V*_d_ = 6 V and *V*_g_ = *V*_s_ = 0 when toggled using intermittent *V*_s_ pulses of ±4 V. **b** Retention time dependence of nonvolatile on/off *I*_g_ with *V*_d_ = 6 V and *V*_g_ = *V*_s_ = 0 when toggled using *V*_s_ pulses of +5 V and −5 V. **c** Drain voltage dependence of drain-source current under various gate voltages for S13 as *V*_s_ = 0 V. The arrows indicate voltage sweeping directions. **d** *V*_g_ dependence of *V*_c_ in **c**.

In addition, the complementary on/off *I*_g_ and *I*_s_ can be controlled using *V*_s_ between −4 and +4 V at various applied *V*_d_ when *V*_g_ = 0, as shown in Fig. 3a for S10, where the initially-on (-off) *I*_g_ (*I*_s_) turns off (on) abruptly below *V*_t2_ (<0) but back on (off) again above *V*_t1_ (>0). There are large misfits at the start and end points in each hysteretic loop. *I*_g_ = −*I*_s_ when *V*_s_ increases from 2 to 3.5 V, thus providing direct evidence of D–S wall contraction from Step 2 into Step 1 through the intermediate Step 3 (Fig. 1a). Both on-to-off and off-to-on *I*_g_ (*I*_s_) switching can be realized during the first *V*_s_ sweeps from 0 → −4 V → 0 and from 0 → 4 V → 0 in Fig. 2b (Supplementary Fig. 4a), respectively; both the on and off states are nonvolatile, as confirmed by the next two repeated cycles. The on-state current increases by more than four times after the first cycle, which appears to correlate with the wall reconstruction after removal of all applied voltages (Supplementary Fig. 4b). Figure 3c shows the nonvolatile on- and off-states when toggled using *V*_s_ pulses between −4 and 4 V at a repeat frequency of 8.2 Hz, where *I*_g_ was accessed intermittently from an oscilloscope at *V*_d_ = 6 V at a repeat frequency of 96 Hz. The switching remains stable versus switching number of up to 240 with an on/off current ratio > 10^4^, as shown in Fig. 4a; and the on- and off-states when toggled using *V*_s_ pulses between −5 and 5 V are stable over retention time of 24 h, as shown in Fig. 4b. During reliability testing, a large body of data from two-terminal LN cells were adopted either at room temperature (Supplementary Fig. 5a–d) or at 85 °C (Supplementary Fig. 6a–d), where G electrodes were omitted for the convenience of electrical characterization and device fabrication. It is believed that the reliability data adopted either in three-terminal transistors or in two-terminal nanodevices are comparative due to the universality of their operation principles on the basis of domain nucleation and growth. The testing results are summarized here: (1) on/off currents in the ratio > 10^4^ are both stable over retention time of >10^6^ s at 20 °C or >10^5^ s at 85 °C; (2) fatigue cycles can highly reach the number of 10^10^ under the inhibited space-charge injection; and (3) operation speeds can be fastened from 330 ns at 500 kV cm^−1^ to <5 ns at 600 kV cm^−1^. Meantime, the diode-like *I*_ds_ current in this study can suppress sneak current paths through the persistent DWs (crosstalk) when using crossbar connection of high-density LN cells (Supplementary Figs 7a–e and 8a, b).

The DW transistor can enable the non-volatile information storage among D–G, G–S, and D–S domains. Though the neutralized D–S wall is more energetically stable than the charged D–G and G–S walls, the write voltage (*V*_ds_ > *V*_c_) is highest. However, once the G electrode is grounded (*V*_g_ = *V*_s_ = 0), the local domain nucleating field between D and G strengthens under a constant *V*_d_; once the head-to-head needle-like domain is nucleated above a critical size between D and G, it can grow throughout the low field G–S region due to the driving force of a depolarization field during the compensation of the increased domain wall energy at the expense of the depolarization energy^23^. In this way, *V*_c_ is reduced. Figure 4c shows *I*_ds_–*V*_d_ curves for S13 at various *V*_g_, where *V*_c_ decreases nearly by one third as *V*_g_ = 0 and reduces further to 4.3 V as *V*_g_ = −1 V with the energy consumption of ~0.15 pJ bit^−1^ (~2*PhwV*_c_). The *V*_c_ can be scaled down almost linearly with *l*_dg_ + *l*_g_ + *l*_gs_, and the addressed cell (*V*_g_ = −1 V) has a much smaller write voltage than other undressed cells (floating), which can increase the reliability of the crossbar architecture (disturbance immunity during the write). Likewise, *V*_c_ can also be reduced through the *E* strengthening between G and S as *V*_g_ > 1.5 V, as indicated from the *V*_g_−*V*_c_ plot in Fig. 4d, since the tail-to-tail needle-like domain once nucleated between G and S can also grow throughout a low field D–G region in the same way.

Remarkably, the nonvolatility in the DW transistors not only reduces gate/source leakage, thus reducing standby power, but also enables programmable nanoprocessor development with computing, memory and addressing capabilities at much faster operating speeds than other charge-trapped FETs^22^.

### Domain wall structure

**Fig. 5:**
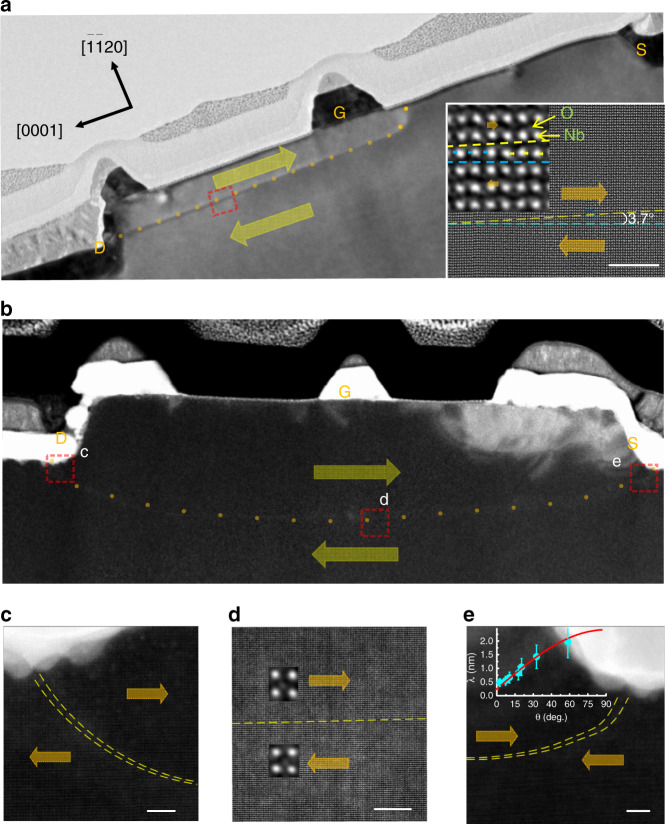
TEM characterization of the domain structure. **a** Low magnification TEM image of thinned S11 lamella viewed along the $\bar 1100$ direction after poling at *V*_d_ = 10 *V* and *V*_g_ = 0 V with S floating, where the dotted and dashed lines delineate a straight D-G wall running along the $000\bar 1$ direction with a tilt angle of 3.7°, as estimated from high-resolution LAADF images of the framed region shown in the insets. **b** Decurved D-S wall in S12 after poling at *V*_d_ = 10 *V* and *V*_g_ = *V*_s_ = 0 V. **c**, **d**, **e** High-resolution LAADF images of the framed regions in **b**, which demonstrate the presences of a charged tail-to-tail wall near D, a neutral wall below G, and a charged head-to-head wall near S; the insets show magnified dipole displacements near the wall regions in panel d and the tilt angle dependence of the wall thickness in panel e. Scale bars: 5 nm. Thick arrows indicate the domain/dipole orientations. The error bars are defined as standard deviation.

The nonvolatile D–G and D–S domain walls in S11 and S12 after voltage poling can be imaged by low-angle annular dark-field scanning transmission electron microscopy (LAADF-STEM), as shown in Fig. 5a–e. Before focused ion beam thinning of the two samples, the D–G and D–S domains were also inspected by in-plane PFM amplitude and phase imaging (Supplementary Fig. 9a, b). LAADF-STEM data were later collected along the [$\bar 1100$] direction (the R3c space group in hexagonal indexes^25^) after optimization of the dose rate, the mechanical instability, and the radiation damage. The heavy Nb atoms appear brighter than the lightweight O atoms, and ferroelectric displacements of Nb cations are inverted horizontally across the domain wall (the dotted line), as indicated by the inset images in Fig. 5a. A straight D–G wall runs slightly beyond G at the mid-height of an etched cell upon the local 180° domain reversal (thick arrows), as indicated by the dotted line in Fig. 5a. Subsequent high-resolution images of the framed region in the insets show the head-to-head wall at an incline angle (*θ*) of 3.7°, which is much higher than the typical ~1° observed in bulk crystals^11^. This increased inclination angle enhances *I*_g_ in Figs. 2 and 3 by more than four orders of magnitude when compared with the 1.2 × 10^−5^–1.0 × 10^−2^ μA μm^−1^ observed in bulk LN crystals^6–11^. When the domain grows throughout the entire cell, a decurved D–S wall then forms at the bottom (Fig. 5b). Subsequent high-resolution images of the framed regions in Fig. 5c–e unambiguously show a charged tail-to-tail wall near D, a neutral wall below G, and a charged head-to-head wall near S. The LAADF-STEM image was fitted with a parametric model in which the column Nb position was derived from the intensity distribution of each atom described as a Gaussian function (Supplementary Figs 10a–d and 11a–c)^12,26^, where off-center displacements of the Nb columns near the ferroelectric domain wall with the thickness *λ* were analyzed using a hyperbolic tangent (tanh) function^27^, as lineated between two dashed lines in Fig. 5c, e. These wall regions are rich with antiparallel dipoles for the D–G wall with a smaller inclined angle in the middle (see the inset in Fig. 5a) but with walls meandering back and forth for the decurved D–S wall with larger inclined angles near the D and S edges (Supplementary Fig. 10b)^12^. *λ* broadens with increasing tilt angle, as illustrated by the plot in the inset of Fig. 5e. These plots can be fitted using a solid line based on the following equation:

3 $${\uplambda} = \lambda _0 + \alpha \cdot (2P\,{\mathrm{sin}}\,\theta ),$$

where *λ*_0_ is the neutral wall thickness, *α* is a coefficient, and 2*P* sin *θ* is the projected polarization charge along the wall. From the fitting, we found that *λ*_0_ = 0.25 nm and *α* = 1.5 × 10^3^ μm^3^ μC^−1^, implying a single unit-cell thickness for a neutral wall (*θ* = 0°) that thickens up to 10 unit cells when *θ* = 90°.

## Discussion

In summary, nonvolatile LiNbO_3_ transistors were fabricated with high complementary on/off source and gate currents that were controlled using either gate or source voltages without subthreshold swings. The working principle of these transistors uses repetitive redirection of the conducting domain walls between D, G, and S upon ultrafast reversal of a collective ferroelectric order parameter. The observed ultrathin wall with high conductivity that could be increased further through wall angle tilt advances the voltage and size scalabilities of all-ferroelectric transistors beyond the limits of traditional MOSFETs. Each cell can perform independent data computation, storage and sensing with superior energy efficiency, stimulating processing-in-memory implementation of neuromorphic computing, general-purpose memcomputing and cybersecurity^1^. However, the digital switches are still premature at present stage when competing to the Si-based transistors: p-MOSFET is lacking for complementary operation in cascaded logic; operation voltages are very large; the hysteresis-free *I*_ds_–*V*_gs_ transfer characteristics are limited in some specific *V*_ds_ as SS = 0; and the nanosecond switching speed between off- and on-states is still insufficient. Some significant improvements are required in logic for the development of next-generation domain wall nanoelectronics technologies.

## Methods

### Nanodevice fabrication

Congruent LN single crystals containing 48.5 mol% Li_2_O with 5 mol% MgO dopant were grown using the Czochralski technique with high-purity (99.99%) Li_2_CO_3_, MgO, and Nb_2_O_5_ powders that were melted at 1250 °C. The crystal was poled into a single domain pattern at 1180–1200 °C at a current density of 8–10 mA cm^−2^ for 30 min. 200-nm-thick poly(methyl methacrylate) photoresist layers were spin-coated onto the surfaces of X-cut LN single crystals; the LN cell sizes were then defined using electron-beam lithography (EBL; JEOL 6300FS). 30-nm-thick Cr mask layers were subsequently deposited by thermal evaporation (NANO 36, Kurt J. Lesker), while any redundant Cr or photoresist layers that lay outside the written area were removed using a lift-off technique. The LN top layer that lay outside the area protected by the Cr mask layer was etched away to depths of 55–70 nm via ion milling using a reactive ion etching system (RIE-10NR, Samco, Japan). Finally, 30-nm-thick Pt top electrode layers were grown by magnetron sputtering (PVD-75, Kurt J. Lesker) at 400 °C; these layers were then etched into the D, G, and S electrodes to contact the LN mesas by repeating the EBL patterning and ion milling processes described above. To mitigate etching damage to the D and S contacts, additional 150-nm-thick electroplated Ni electrodes with widths of ~300 nm were fabricated above the Pt to strengthen the electrode contacts. All transistors were checked via planar-view SEM images (Sigma HD, Zeiss).

### Phase-field simulations

The spatiotemporal evolution of the spontaneous polarization *P*_*i*_ was determined using the time-dependent Ginzburg-Landau (TDGL) equations^28^:

4 $$\frac{{\partial P_i({\mathbf{r}},t)}}{{\partial t}} = - L\frac{{\delta F}}{{\delta P_i\left( {{\mathbf{r}},t} \right)}}{\mathrm{(i}} = {\mathrm{1,2,3),}}$$

where *L* is the kinetic coefficient related to the domain mobility, $\frac{{\delta F}}{{\delta P_i\left( {{\mathbf{r}},t} \right)}}$ is the thermodynamic driving force for domain evolution, *F* is the total free energy, **r**(*x*_1_, *x*_2,_ *x*_3_) is the spatial vector, and *t* is time. The total free energy is given by the following equation:

5 $$F = {\int} {(f_{{\mathrm{Land}}} + f_{{\mathrm{grad}}} + f_{{\mathrm{elec}}})} dV,$$

where *f*_Land_, *f*_grad_, and *f*_elec_ are the Landau energy, gradient energy, and electrical energy densities, respectively. The Landau energy density can be expressed as $f_{{\mathrm{Land}}} = \alpha _1P_3^2 + \alpha _{{\mathrm{11}}}P_3^4 + \alpha _2P_1^2$ (refs. ^29,30^), where *α*_1_, *α*_11_, and *α*_2_ are the dielectric stiffnesses and the higher-order dielectric stiffnesses, respectively. The gradient energy density is related to the domain wall energy such that $f_{{\mathrm{grad}}} = \frac{1}{2}{G _{ijkl}}{P_{i,j}}{P_{k,l}}$, where *P*_*i*,*j*_ is the spatial derivative of the *i*th component of polarization vector *P*_*i*_ with respect to the *j*th coordinate and *G*_*ijkl*_ represent the gradient energy coefficients. The electrostatic energy can be expressed as $f_{{\mathrm{elec}}} = - \frac{1}{2}\kappa _cE_iE_i - E_iP_i$, where *κ*_*c*_ is the dielectric permittivity of the background materials. The electric field is calculated from $E_i = - \frac{{\partial \varphi }}{{\partial x_i}}$, where *φ* is the electrical potential. It was obtained by solving the Poisson equation of ∇^2^*φ* = 0 using the specified electric potentials at the D, G, and S electrodes of the LN transistor. An open-circuit boundary condition was imposed on the top free surface area without the electrode. The elastic energy term is neglected in Eq. (5) based on the assumption that the elastic energy makes only a small contribution to the final domain wall configuration. To solve the governing equations above in the real space, a nonlinear multi-field coupling finite-element method was used. All the material constants used in the simulations are *α*_1_ = −1.0 × 10^9^ C^−2^ m^2^ N, $\alpha _2 = {\mathrm{0}}{\mathrm{.9725}} \,\times {\mathrm{10}}^9\,{\mathrm{C}}^{ - 2}\,{\mathrm{m}}^2\,{\mathrm{N}}$, $\alpha _{11} = {\mathrm{0}}{\mathrm{.9025}} \,\times {\mathrm{10}}^9\,{\mathrm{C}}^{ - 4}\,{\mathrm{m}}^6\,{\mathrm{N}}$, $G_{{\mathrm{11}}} = G_{{\mathrm{44}}} = {\mathrm{0}}{\mathrm{.4}} \,\times \left| {\alpha _1} \right|$, and $\kappa _c = {\mathrm{10}} \,\times \kappa _0 = {\mathrm{10}} \,\times {\mathrm{8}}{\mathrm{.85}} \,\times {\mathrm{10}}^{ - 12}\,{\mathrm{F}}\,{\mathrm{m}}^{ - 1}$ (refs. ^25,28^). For convenience of the calculation, the material parameters were normalized^31^. The simulations were performed using a two-dimensional system, and four-node elements with a size of 1 nm^2^ were used to model the LN structure. To model the semi-infinite LN single crystal substrate, the charge densities on the right and left edges of the LN substrate were set to be +*P* and −*P*, respectively. Partially reversed polarizations are assumed as the initial conditions to initiate the domain evolution process in the LN structure under application of various values of *V*_d_, *V*_g_, and *V*_s_. The final equilibrium domain structures were obtained under the applied voltages as shown in Fig. 1a (Supplementary Fig. 1a–c).

### PFM and LAADF-STEM characterization

The domain patterns obtained after poling using positive and negative voltages were inspected via in-plane PFM amplitude and phase imaging (Icon, Bruker) using a contact PtIr-coated silicon tip with radius of ~20 nm, a force constant of 2.8 N m^−1^, and an AC amplitude of 0.5 V at 210 kHz. The LN surface was later coated with a protective Pt layer (∼35 nm thick). A cross-sectional specimen was cut and thinned using a dual-beam focused ion beam/scanning microscopy (Helios G4 UX, Thermo Fisher Scientific, USA) system with Ga-ion acceleration voltages ranging from 2 to 30 kV followed by ion-milling (Gatan 691, Gatan, USA) at voltages ranging from 1.5 to 0.5 kV to remove the damaged layer. A 200 kV JEM-2100F (JEOL Ltd., Japan) microscope was used to perform the LAADF-STEM observations.

### Electrical characterization

All current–voltage curves were measured using an Agilent B1500A semiconductor analyzer operating in voltage-sweep mode. The sweep times were 1 s when using a current amplification range of 1 μA with instrumental off-current resolutions of 20 pA. For domain switching testing, two square test pulses with rise times of 2 ns were supplied to D and G (S) using a two-channel Agilent 81110A pulse generator. The steady-state on-and-off *I*_s_ (*I*_g_) current transient behavior with time was observed directly using a four-channel oscilloscope (LeCroy HDO6054, USA) in series with the *S* (*G*) with 12-bit voltage resolution and a 1 GHz bandwidth. During the nanosecond-scale domain-switching period, the internal resistance of the oscilloscope in series with the sample was adjusted to 50 Ω to realize a short circuit RC time constant, and was later adjusted to 1 MΩ (100 kΩ) to enable read-out of on- and off-currents with 0.2 nA (1 nA) resolution limits as shown in Figs. 2e and 3c, respectively.

## Supplementary information

Supplementary Information

Peer Review File

## Data Availability

The authors declare that all data supporting the findings of this study are available within the paper and its supplementary information files.
