# Supplementary material for: Nonvolatile ferroelectric field-effect transistors
Source: Nat Commun. 2020 Jun 4;11:2811. doi: 10.1038/s41467-020-16623-9 (PMC7272614; doi:10.1038/s41467-020-16623-9)
Supplement: Supplementary file 1 — Supplementary Information [file 41467_2020_16623_MOESM1_ESM.pdf]

Supplementary Information for

Nonvolatile ferroelectric field-effect transistors

Chai et al.

Supplementary Note 1. Phase-field simulations

Supplementary Note 2. Hysteretic field-effect transistors

Supplementary Note 3. Electrode contacts and large wall currents

Supplementary Note 4. Wall reconstruction

Supplementary Note 5. Reliability testing

Supplementary Note 6. Method to estimate DW thicknesses

Supplementary Note 1. Phase-field simulations

The spatiotemporal evolution of the spontaneous polarization was determined using the time-dependent Ginzburg-Landau equations. Spatial electric potential distribution for S2 in Fig. 1a in the main text was obtained by solving the Poisson equation of $\nabla^2\phi = 0$ from which the local electric field was calculated from $E_i = -\partial\phi/\partial x_i$, as shown in Supplementary Figure 1a-c (right panels). The geometrical sizes for S2 can be found in Table 1 in the main text. The phase-field simulations in Supplementary Figure 1a-c (left panels) exhibit repetitive redirection of the conducting domain walls against V_g between D, G and S in three steps when V_d = 8.5 V and V_s = 0. As V_g = -8 V, the head-to-head wedged domain initiating from D grows up below G with the length of 355 nm and the thickness of 46 nm, as shown in Supplementary Figure 1a (left panel). The aspect ratio for the needle-like domain is 7.7 that is generally constant irrespective of the geometrical sizes[1]. In this sense, the smaller l_{dg} implies the thinner D-G domain, and the etching depth (h) of the device should be larger than the thickness of the D-G domain. The insets in Supplementary Figure 1a-c show the wall thicknesses of 0.72–1.06 nm when θ = 0–8.4^o, roughly in agreement with the TEM estimations in Fig. 5e in the main text. The domain can grow throughout the whole cell (l_{dg}+l_g+l_{gs}) as V_g = 0, as shown in Supplementary Figure 1b, which contracts into Supplementary Figure 1a again through an intermediate Step 3 in Supplementary Figure 1c as V_g = -5 V. Meantime, the simulations also show the rounded corners of the D-S wall near D and S in Supplementary Figure 1c (left panel), in prediction of a decurved D-S wall in Fig. 5b in the main text.

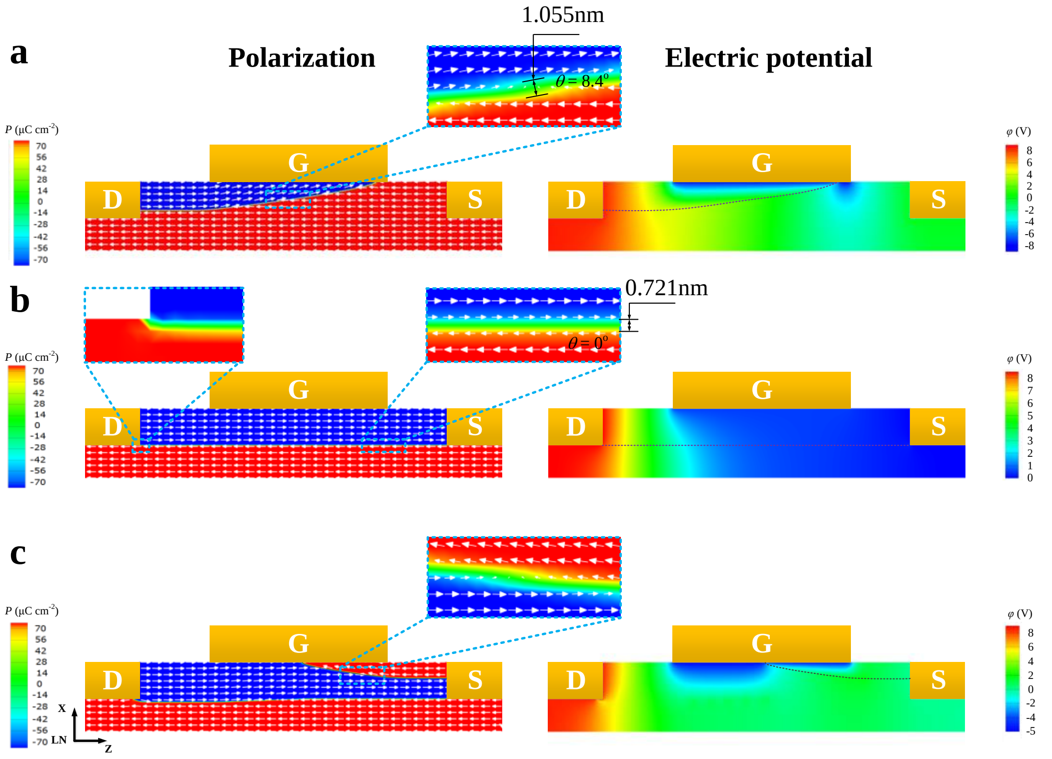

Supplementary Figure 1. Detailed domain structures and electric potential distributions in Fig. 1a in the main text. **a, b, c** The phase-field simulations of domain structures (left panels) and electric potential distributions (right panels) for S2 with the increase of V_g from -8 V→ 0 V → -5 V in three steps as $V_g < V_{t1}$, $V_g > V_{t1}$, and $V_g < V_{t2}$, respectively, where V_d = 8.5 V, V_s = 0, V_{t1} = -4.70 V, and V_{t2} = -4.72 V. The insert figures magnify domain walls in the framed regions. All arrows show domain orientations, and the dashed lines in right panels indicate the DW locations.

Supplementary Note 2. Hysteretic field-effect transistors

The planar SEM image in Supplementary Figure 2a (left panel) shows that l_{dg} and l_{gs} for S8 are as long as 203 nm and 280 nm, respectively, to avoid D-G and G-S domain switching during sweeping of V_g between –8 V and 2 V. From this image, it is clear that the D and S electrode contacts entirely cover the side slopes of an etched LN cell. The right panel in Supplementary Figure 2a shows the hysteretic I_{ds}-V_g sweeping curves at various applied V_d values when $V_s = 0$ after creation of the nonvolatile D-S wall at 32 V for S8. I_{ds} is obviously on within the positive voltage range but is off below −6 V with a hysteretic voltage of ~1.8 V between the forward and backward sweeping curves, indicating the major carrier accumulation/depletion mechanism that occurs within an n-type D-S conduction channel. When the heights of the D and S electrode contacts relative to the LN mesa (~65 nm) are halved for S13, as shown in the atomic force microscopy (AFM) and cross-sectional SEM (inset) images (left panel in Supplementary Figure 2b), the hysteresis is suppressed considerably, as confirmed by the I_{ds}-V_g sweeps shown in the right panel. Therefore, the I_{ds}-V_g sweeping hysteresis seems to be correlated with charge injection into the LN near-surface damage layer during device etching; reduction of the heights of the D and S electrode contacts could reduce this risk, although the wall current density was reduced by nearly half because of the poor electrode contacts.

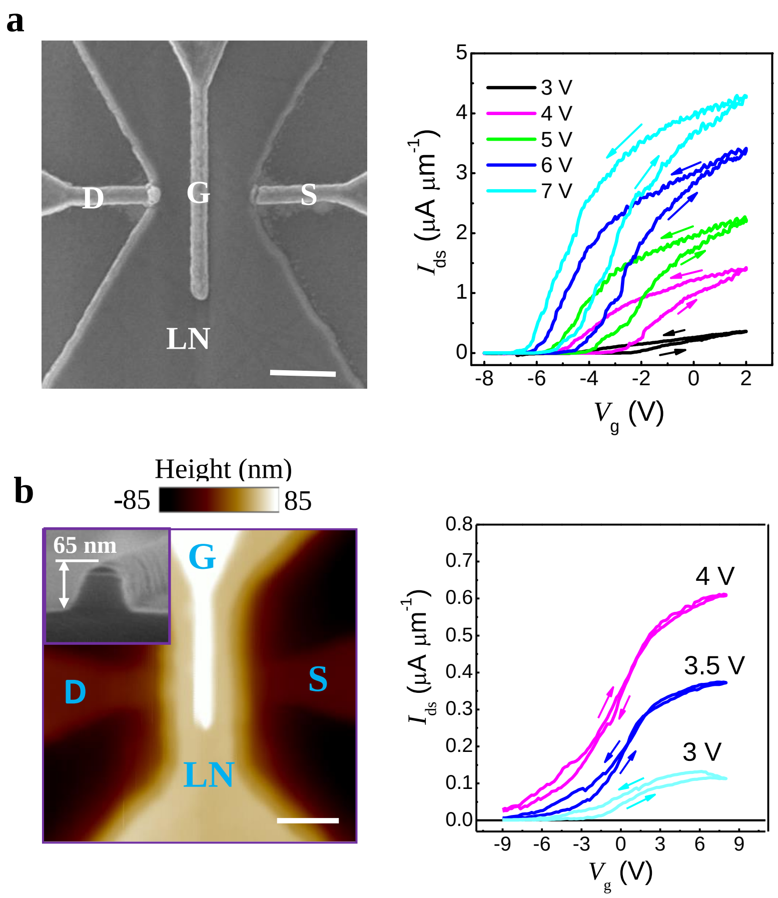

Supplementary Figure 2. Traditional field-effect transistors. **a** Planar SEM image of three-terminal LN transistor for S8 (left panel). The scalar bar: 400 nm. The right panel shows the hysteretic I_{ds}-V_g sweeping curves at $V_s = 0$ under application of various V_d across the unchanged D-S wall channel without invoking D-G and G-S wall formation. **b** AFM image (left panel) of a transistor with D and S electrode contact heights lowered to the LN mesa for S13. The LN etching depth is shown by an inset cross-sectional SEM image. The scalar bar: 200 nm. The lowered heights of the D and S electrode contacts minimize the hysteresis of the I_{ds}-V_{gs} curves under application of various V_d (right panel). All arrows indicate voltage sweeping directions.

Supplementary Note 3. Electrode contacts and large wall currents

All ferroelectric transistors can operate correctly during repetitive switching cycles. Supplementary Figure 3a shows 100 I_{ds}-V_g sweeps in semi-logarithmic plots between −9 V and 1 V for S2 when V_d = 8.5 V and V_s = 0. The on-and-off state switching is abrupt (SS = 0), with a high on/off ratio of ~10^4, although V_{t1} and V_{t2} both have broad dispersions (~0.8 V) over the switching cycles.

The wall current is normally in the 1.2×10^{-5}–1.0×10^{-2} μA μm^{-1} range in bulk LN single crystals[2,3] but increases by more than 10,000 times in our nanodevices (Supplementary Figure 3b–d). The maximum wall currents here are limited by the poor Pt electrode contacts to the LN cell; these contacts were easily damaged during etching. To mitigate this damage, 150-nm-thick electroplated Ni electrodes were fabricated above the Pt surface, as shown in the planar-view SEM image of a two-terminal LN cell in Supplementary Figure 3b. Subsequent double current-voltage measurements in Supplementary Figure 3c showed the wall current as high as ~110 μA μm^{-1} at 656 kV cm^{-1} with a coercive field of 492 kV cm^{-1} during the first sweep, while the coercive field stabilized at 284 kV cm^{-1} after three runs. Finally, 120 I_{ds}-V_{ds} sweeps between −4 V and +4 V in Supplementary Figure 3d, indicated that the wall current was as high as 36 μA μm^{-1} at 4 V with a negligible coercive voltage dispersion over the sweep cycles. The wall current is space-charge limited but can be fitted using a power law with the coefficient of 2.4 (see ref. 4).

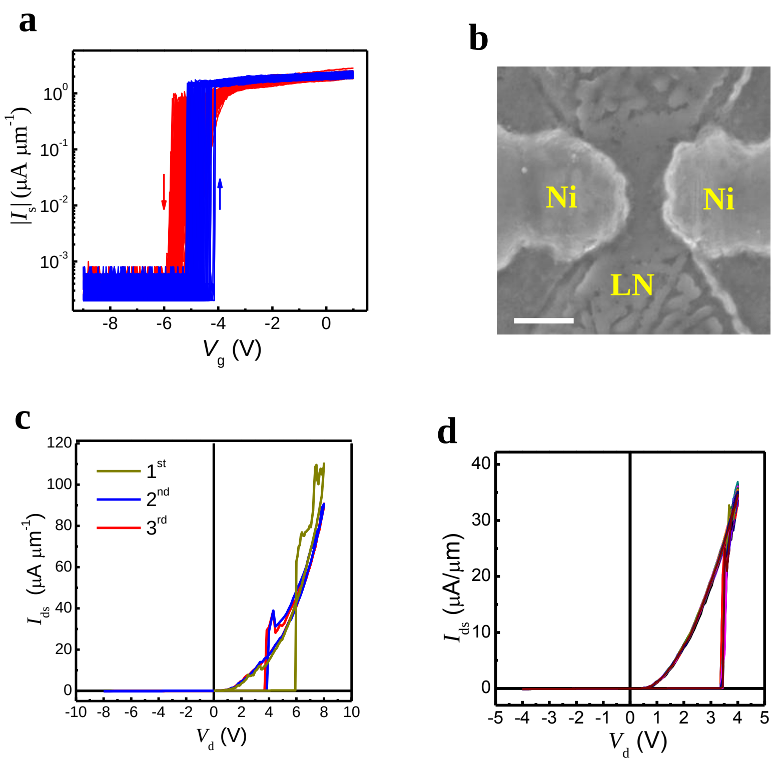

Supplementary Figure 3. Good electrode contacts and large wall currents. **a** Semi-logarithmic plots of 100 $|I_s|$-V_g sweeps with V_d = 8.5 V and V_s = 0 for S2. **b** Planar-view SEM image of a two-terminal LN cell with good contacts to two electroplated Ni electrodes. The scalar bar: 200 nm. **c** The three initial I_{ds}-V_d curves. **d** 120 sweeps of the I_{ds}-V_d curves between −4 V and +4 V.

Supplementary Note 4. **Wall reconstruction**

In the V_s-controlled nonvolatile transistor, I_g turns off permanently upon sweeping of V_s from 0 → −4 V→ 0 but turns on again upon sweeping from 0 → 4 V→ 0 (Fig. 3a–c in the main text); this is in contrast to the behaviour of the complementary I_s in Supplementary Figure 4a, which responds in the opposite way. The sketches shown in Supplementary Figure 4b assume that four processes are required for wall reconstruction during V_s cycling. Initially, the head-to-head D-G wall in process 1 stabilizes over time after removal of all the applied voltages. At this stage, I_g is on but I_s is off. This process remains nearly unchanged as V_d increases from 0 to 6 V. Next, when V_s sweeps downward below −1.3 V, the domain grows throughout the cell during formation of a D-S wall to turn on (off) I_s (I_g) in process 2. When V_s sweeps upward to exceed 2.4 V, the partial G-S domain in process 3 is reversed to generate an I_{sg} that is much higher than I_{ds}, as inferred from Supplementary Figure 4a (lower panel). At this moment, $I_s \approx -I_g = -1.76$ μA μm^{-1} in comparison to Fig. 3b (lower panel) in the main text. With further increases in V_s above 3.4 V, comprehensive G-S domain switching occurs during process 4 to turn on I_{dg} while also turning off I_{ds}. However, the D-G wall at the centre is nearly neutral ($\theta = 0$) and is less conductive than the wall in process 1 ($\theta = 3.7^o$). This is the reason why I_g is much lower during the first I_g-V_s sweep (lower panel in Fig. 3b in the main text), and $|I_s|$ does not decrease to 0 when V_s = 4 V (see the first sweep in Supplementary Figure 4a) because of the leakage current from the D-G wall at the right corner close to S. Once V_d and V_s are both reduced to 0, the decurved D-G wall in process 4 retracts into a charged D-G wall in process 1 to reduce the depolarization energy of the triangular charged wall on the right. With domain retraction, I_g increases by more than four times during the second and third sweeps shown in Fig. 3a–c in the main text, and I_s returns to 0 when

V_s = 4 V (see the second and third sweeps in Supplementary Figure 4a) because of the enhanced distance between the D-G wall and S, which reduces the leakage current.

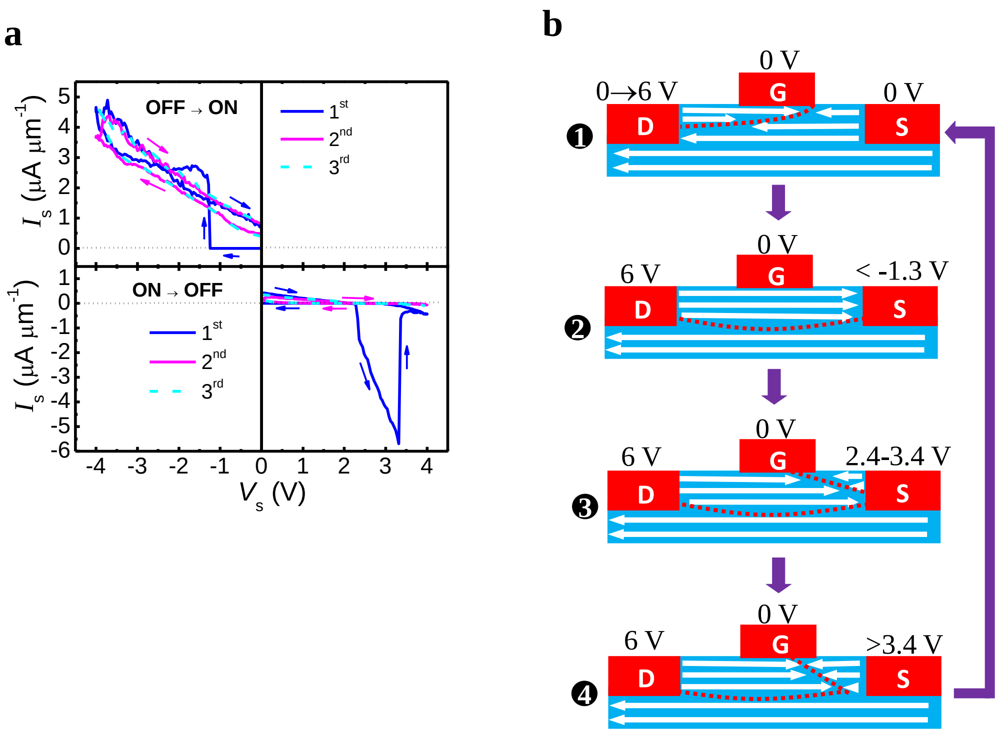

Supplementary Figure 4. Wall reconstruction for V_s-controlled transistors. **a** Off-to-on and on-to-off I_s when toggled using the first sweep of V_s, which varies either from 0 → −4 V→ 0 (upper panel) or from 0 → 4 V→ 0 (lower panel), with V_d = 6 V and V_g = 0 for S10. Each transition is nonvolatile, as confirmed by the following two repeated cycles. **b** Sketches for D-G wall reconstruction during V_s sweeping from 0 → −4 V→ 0→ 4 V→ 0.

Supplementary Note 5. Reliability testing

Non-volatile three-terminal LN transistors can perform *in situ* data computing, storage, and sensing operations with high reliability. During the reliability testing, we selected a large body of data from two-terminal cells integrated with D and S electrodes for the comparison of on and off I_{ds} during read and write operations under various conditions, where G was omitted for the convenience of electrical characterization and device fabrication. Supplementary Figure 5a shows retention time dependences of on- and off-currents at 4 V after two write voltages of + 10 V and -10 V for a two-terminal memory cell with the sizes of $w \times l \times h$ = 150×150×60 nm^2 at room temperature. The on/off current ratio highly reaches 10^6 that is stable over laboratory time of 10 days, implying good retention of the data once written within the device. Supplementary Figure 5b shows switching time dependence of readout current at 200 kV cm^{-1} under various applied electric fields, where the switching time can be shortened from 330 ns to <5 ns with the increase of E from 500 kV cm^{-1} to 600 kV cm^{-1}. In principle, domain switching initiates from the reversed domain forward growth at time τ_1 in nucleus number N from the LN interface near D, and the domains would later coalesce via wall sideways motion until N = 1 after time τ_2 (ref. 5). To follow this process, I_{ds} switches from an off current (I_{off}) to an on current (I_{on}) at τ_1 that decreases up to τ_2 as the domain coalesces. τ_1 < 5 ns when E = 600 kV cm^{-1}, which can be described using Merz's law[6], $\tau_1 = \tau_0 \exp\left(\frac{E_a}{E}\right)$, where E_a is the activation field and τ_0 is the shortest time of ~10^{-13} s (ref. 7). If $I_{ds} \propto N$ with a domain coalescence rate of $\frac{d(N-1)}{d(t-\tau_1)} = -\frac{N-1}{\tau_2}$ ($t \geq \tau_1$), we obtain

$$I_{ds} = \begin{cases} I_{off} \quad (t < \tau_1) \\ I_{on}\left[1 + (N-1)\exp\left(-\dfrac{t-\tau_1}{\tau_2}\right)\right] \quad (t \ge \tau_1) \end{cases} . \tag{1}$$

The solid lines in Supplementary Figure 5b represent the best fittings of the data in accordance with Supplementary Equation 1, from which we extracted N = 2 and E_a = 6.9 MV cm^{-1}.

Subsequent fatigue testing under application of square pulses with bipolar voltages of ±10 V at 1 MHz in full duty conditions shows the reduction of V_c from 7.6 V to 4.6 V after 10^7 cycles, as inferred from *I-V* curves in Supplementary Figure 5d. This could be correlated with the asymmetrical charge injection that preferably occurs at +10 V. The charge injection can induce an imprint field to reduce V_c rather than prediction of any polarization fatigue. After optimization of these bipolar voltages to +8 V/−10 V, the readout on-/off-currents at 4 V are stable for cycling numbers of up to 10^{10}, as shown in Supplementary Figure 5c, where V_c is eventually balanced at 5.7 V (Supplementary Figure 5d). This predicts good endurance of the device, once the charge injection is suppressed.

With the elevated temperature up to 85°C, the high reliability remains for the LN cells integrated on Si substrates, as shown in Supplementary Figure 6a-d. During the tests, 500 nm-thick defect-free monodomain LN thin films were cleaved from bulk single crystals and bonded to SiO_2-passivated Si wafers to form LN-on-insulator (or LOI) structures[8-10]. Supplementary Figure 6a shows the SEM photograph of a cell in sizes of $w \times l \times h$ = 83×41×40 nm^3. Supplementary Figure 6b shows the *I-V* curves swept between – 6 V and 6 V and between 0 and 6 V after two poling voltages of – 6 V and 6 V, respectively, where the off-to-on current jump occurs at 3.6 V (V_c). Supplementary Figure 6c shows retention time dependence of readout current at 3 V for the cell after write voltages of +6 V and -6 V, respectively, where the large on/off

ratio of $>10^4$ is stable over the retention time of 24 h. Supplementary Figure 6d shows pulse width dependence of readout current at 3 V after different write voltages, where domain switching time is shortened from 3200 ns at 4 V to <10 ns at 8 V and obeys the Merz's law[6].

The diode-like I_{ds} current can suppress sneak current paths through the persistent DWs when using crossbar connection of high-density LOI cells, as sketched in Supplementary Figure 7a. Supplementary Figure 7b shows the SEM photograph of 16×16 arrays of LOI cells ($w \times l \times h$ = 240×300×70 nm^3) before the interconnection of top electrodes. Supplementary Figure 7c-e exhibits the statistical *I*-*V* curves, on and off currents at 6 V, and coercive voltages over 100 cells. The dispersions either in on/off currents or coercive voltages largely depend on poor electrode contacts and variations of the cell sizes patterned using EBL with overlayer accuracies of 20 nm. For the crosstalk inspection, we fabricated another 8×8 arrays of LOI cells, where the cell 0 was surrounded by the neighbouring cells 1-8 , as indicated from the SEM photograph in Supplementary Figure 8a. Subsequent measurements of *I*-*V* curves between -15 V and 15 V in Supplementary Figure 8b show nearly constant on/off currents irrespective of step-by-step electrical opening of the neighbouring cells at 15 V. V_c decreases by 20% during cycling that eventually levels off at 9.0 V. The high on/off ratio of $\sim 10^4$ highlights the superiority of diode-like DW currents that can inhibit crosstalk from the neighbouring cells.

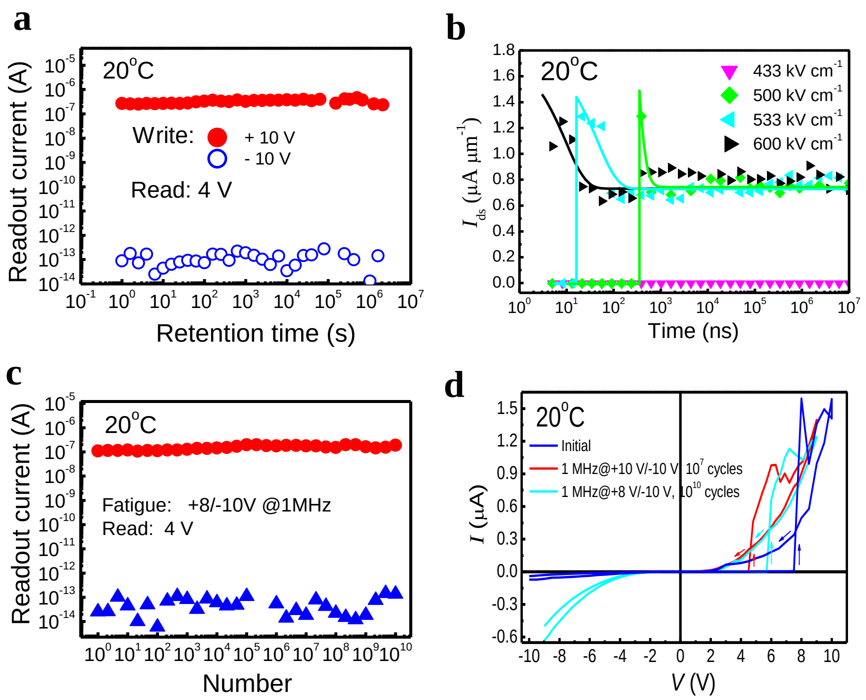

Supplementary Figure 5. Reliability testing at 20°C. **a** Retention time dependences of on- and off-currents at 4 V after different write voltages for a two-terminal memory cell with $w \times l \times h$ = 150×150×60 nm^2. **b** Switching time dependence of readout current at 200 kV cm^{-1} under various applied electric fields, where the solid lines are fitted according to Supplementary Equation 1. **c** Write number dependences of on-/off-currents at 4 V under bipolar switching voltages of +8 V/−10 V at 1 MHz. **d** *I*-*V* curves after different fatigue cycles.

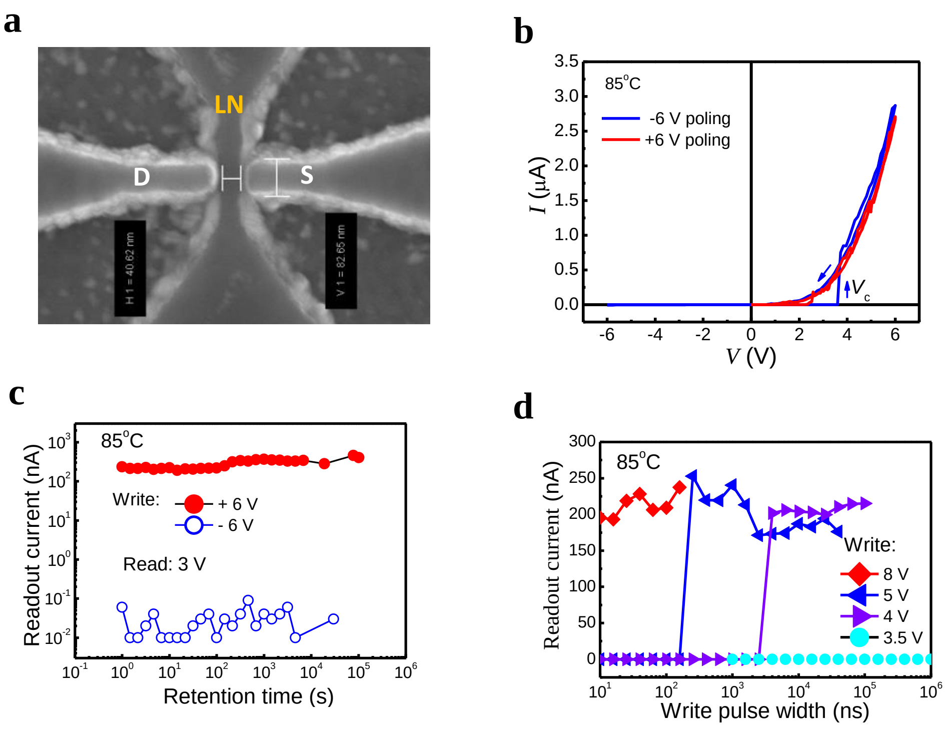

Supplementary Figure 6. Reliability testing at 85°C. **a** The SEM photograph of an LOI cell in sizes of $w \times l \times h$ = 83×41×40 nm^3. **b** *I-V* curves between -6 V and +6 V and between 0 and 6 V for the cell after poling voltages of -6 V and + 6V, respectively. **c** Retention time dependences of on- and off-currents at 3 V for the cell after different write voltages. **d** Pulse width dependence of readout current at 3 V after different write voltages.

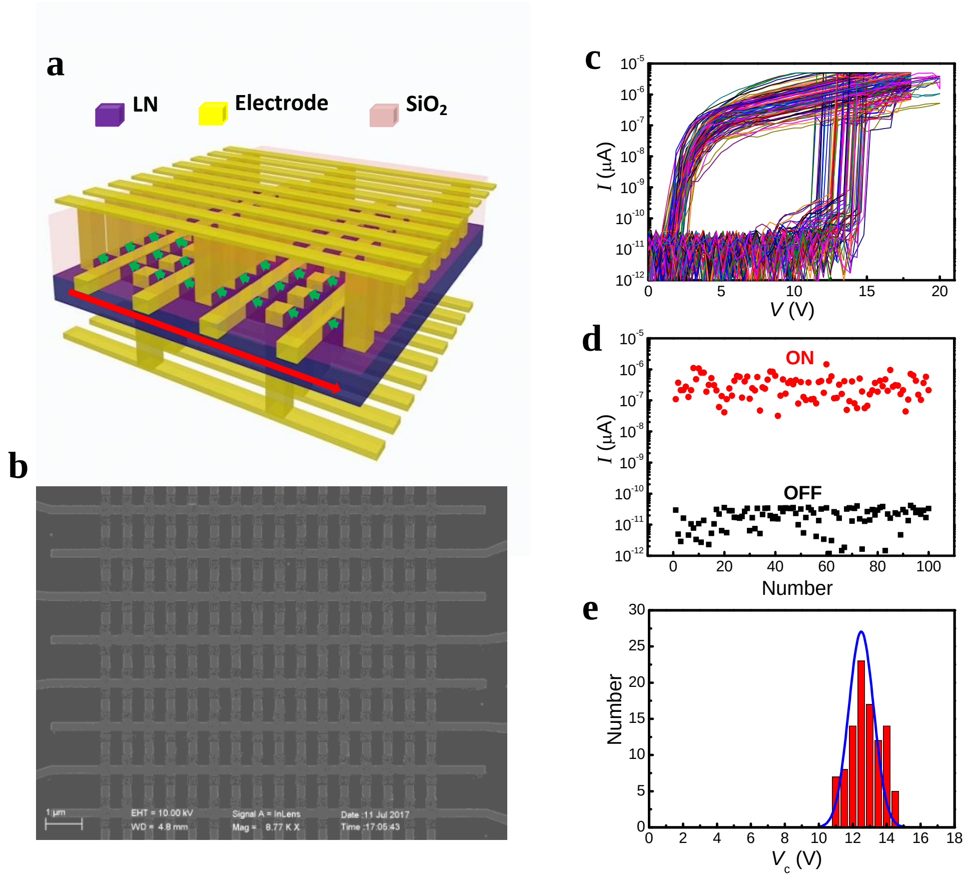

Supplementary Figure 7. Crossbar architecture. **a** The sketch of crossbar architecture. **b** The SEM photograph of 16××16 arrays of LOI cells in sizes of $w \times l \times h$ = 240×300×70 nm^3 before the interconnection of top electrodes. **c**, **d**, **e** Statistical distributions of *I-V* curves, on and off currents at 6 V, and coercive voltages over 100 cells. The compliance is set to be 5 μA to protect the devices against electrical damage during *I-V* measurements. Thick arrows indicate domain orientations. The solid line in panel is fitted according to the Gaussian function.

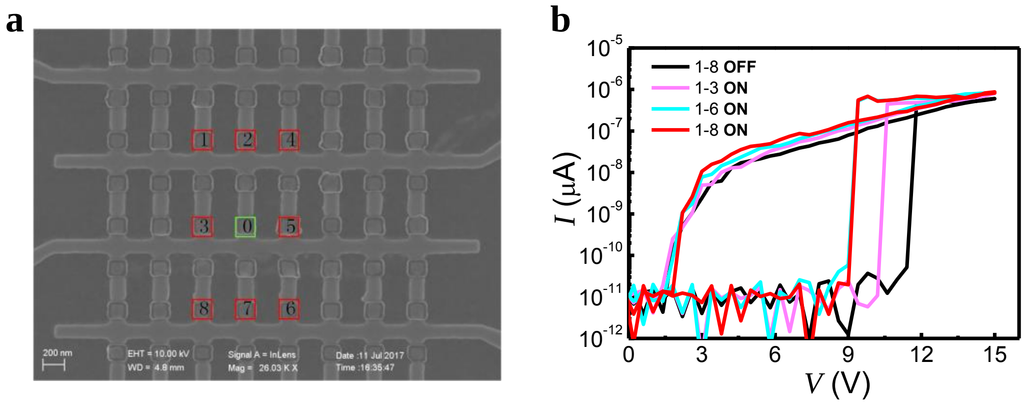

Supplementary Figure 8. Crosstalk. **a** The SEM photograph of 8×8 arrays of LOI cells in sizes of $w \times l \times h$ = 200×200×70 nm^3 before the interconnection of top electrodes. **b** The evolution of *I-V* curves for the cell 0 with electrical opening of neighbouring cells 1-8 in panel a after poling at 15 V.

Supplementary Note 6. Method to estimate DW thicknesses

The nonvolatile D-G and D-S domains in S11 and S12 after voltage poling can be imaged by in-plane PFM magnitude and phase images, as shown in Supplementary Figure 9a, b, respectively. After sample poling at V_d = 10 V and V_g = 0 V with S floating for S11, the black coloured region between D and G has a 180° phase shift in contrast to the peripheral unswitched region in white colour, as shown in Supplementary Figure 9a (right panel). Meantime, the black thick-line wall regions were observed from the PFM amplitude image (left panel), and the reversed domain was much wider than the D electrode width, indicative of the wall sideways motion outside the electrode covering area. As the sample was poled at V_d = 10 V with V_g = V_s = 0 V for S12, the reversed domain between D and G can grow up throughout the entire mesa-like region between G and S, as shown in Supplementary Figure 9b. The driving force for the penetrating needle-like domain growth between D and G over the zero-field region between G and S originates from a depolarization field during the compensation of the increased domain wall energy at the expense of the depolarization energy[5].

LAADF-STEM data were later collected along the $[\bar{1}100]$ direction for the two thinned samples. Supplementary Figure 10a shows the high-resolution LAADF-STEM cross-sectional image for S12 at the region near D, where two dashed lines lineate the wall thickness. The LAADF-STEM image was fitted with a parametric model in which the column Nb position (x, y) was derived from the intensity distribution of each atom described as a Gaussian function[11,12], as shown in Supplementary Figure 10b. All Nb ion positions at the bottom (indicated by the left arrow) were used as the reference, which makes the opposing ferroelectric displacements of Nb ions (indicated by the right arrow) at the top very clear.

Off-center displacement of the Nb columns near the ferroelectric domain wall with the thickness λ were analyzed using a simple parametric model[13]:

$$x = P_s \tanh\left(\frac{y - y_0}{0.5\lambda}\right) + x_0 , \qquad (2)$$

where P_s is a constant displacement and (x_0, y_0) is the local wall position. The two solid lines in Supplementary Figure 10c are the best fits of the data for Nb columns at points A and B. From the fits, λ at each Nb column was extracted and was plotted against x in Supplementary Figure 10d. The wall between two dashed lines in Supplementary Figure 10b has the meandering nature of the interface along the viewing direction, which agrees with the previous observations[11].

Using the same method, we also mapped the Nb ion displacements shown in Supplementary Figure 11b for the framed region in Supplementary Figure 11a, from which the inclined angle of the D-G wall in S11 was derived to be 3.7^{o} with the mean wall thickness of 0.41 nm, as fitted by a solid line according to the Gaussian distribution function (Supplementary Figure 11c).

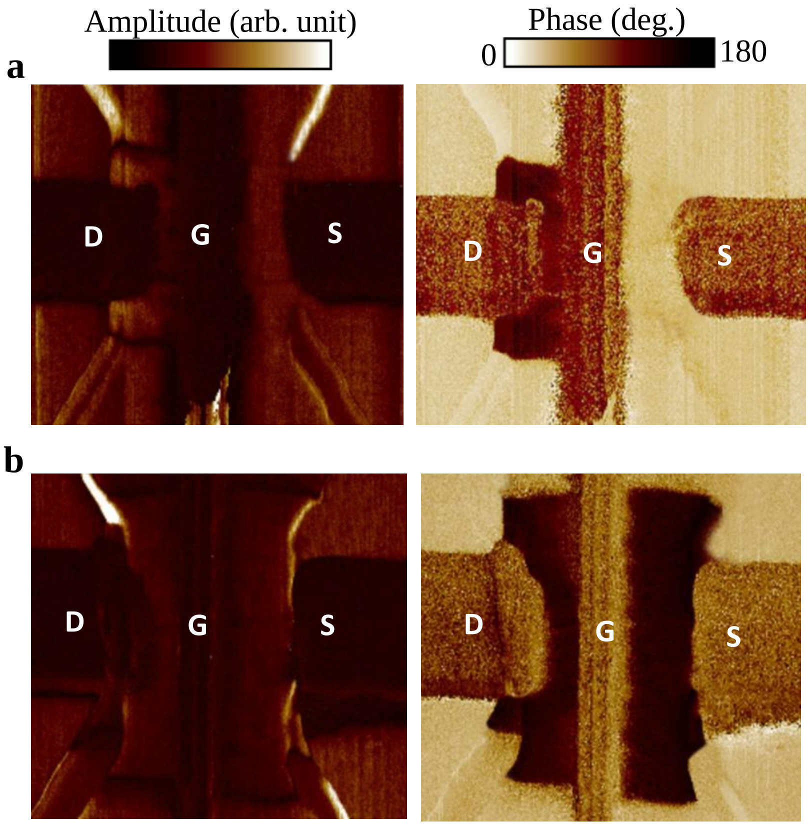

Supplementary Figure 9. PFM imaging of D-G and D-S domains. **a**, **b** In-plane PFM magnitude and phase images after sample poling for creation of the nonvolatile D-G and D-S walls at V_d = 10 V and V_g = 0 V with S floating for S11 and at V_d = 10 V with V_g = V_s = 0 V for S12, respectively.

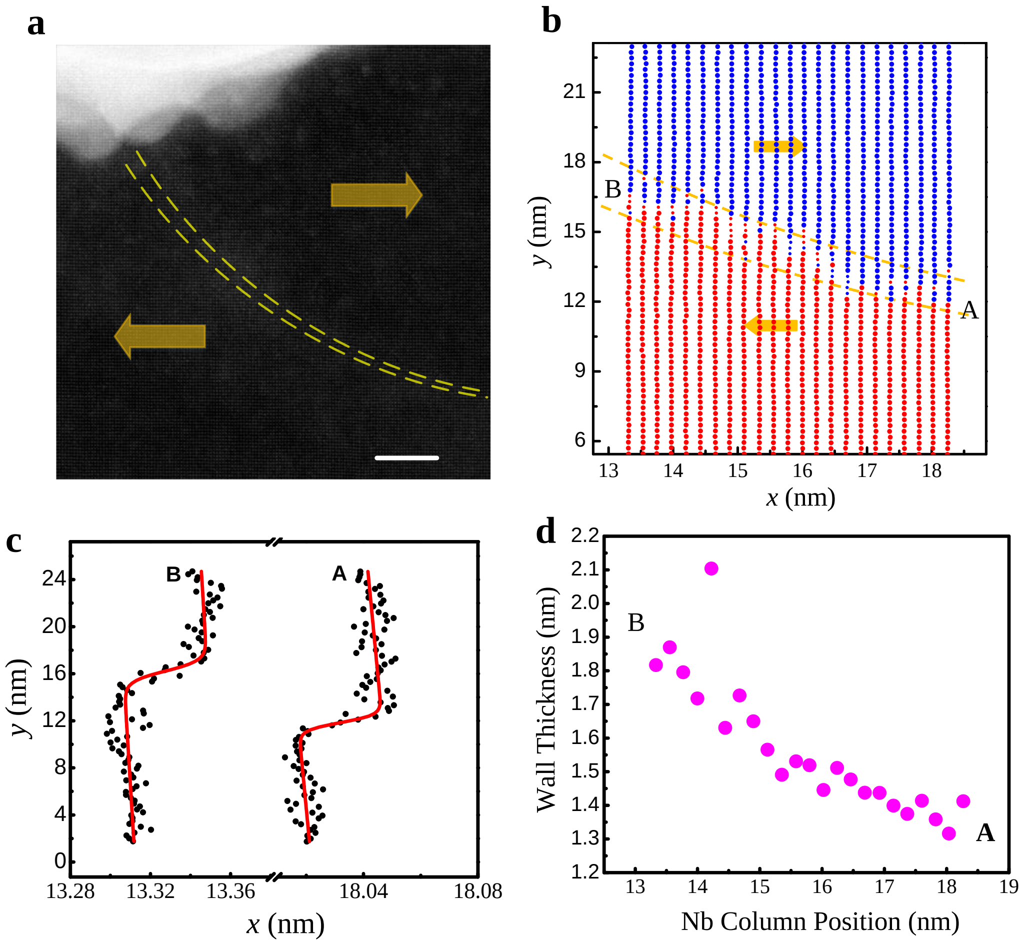

Supplementary Figure 10. Domain wall thickness near the edge. **a** High-resolution LAADF-STEM cross-sectional image for S12 near D viewed along the $[\bar{1}100]$ direction in Fig. 5c in the main text, where two dashed lines lineate the wall thickness. **b** Red and blue dots correspond to the fitted Nb column positions for the framed region in panel a with opposite ionic displacements (thick arrow directions) across the wall region. **c** Solid-line fitting of projected two-dimensional coordinates for two Nb columns at points A and B in panel b. **d** Nb column position dependence of wall thickness.

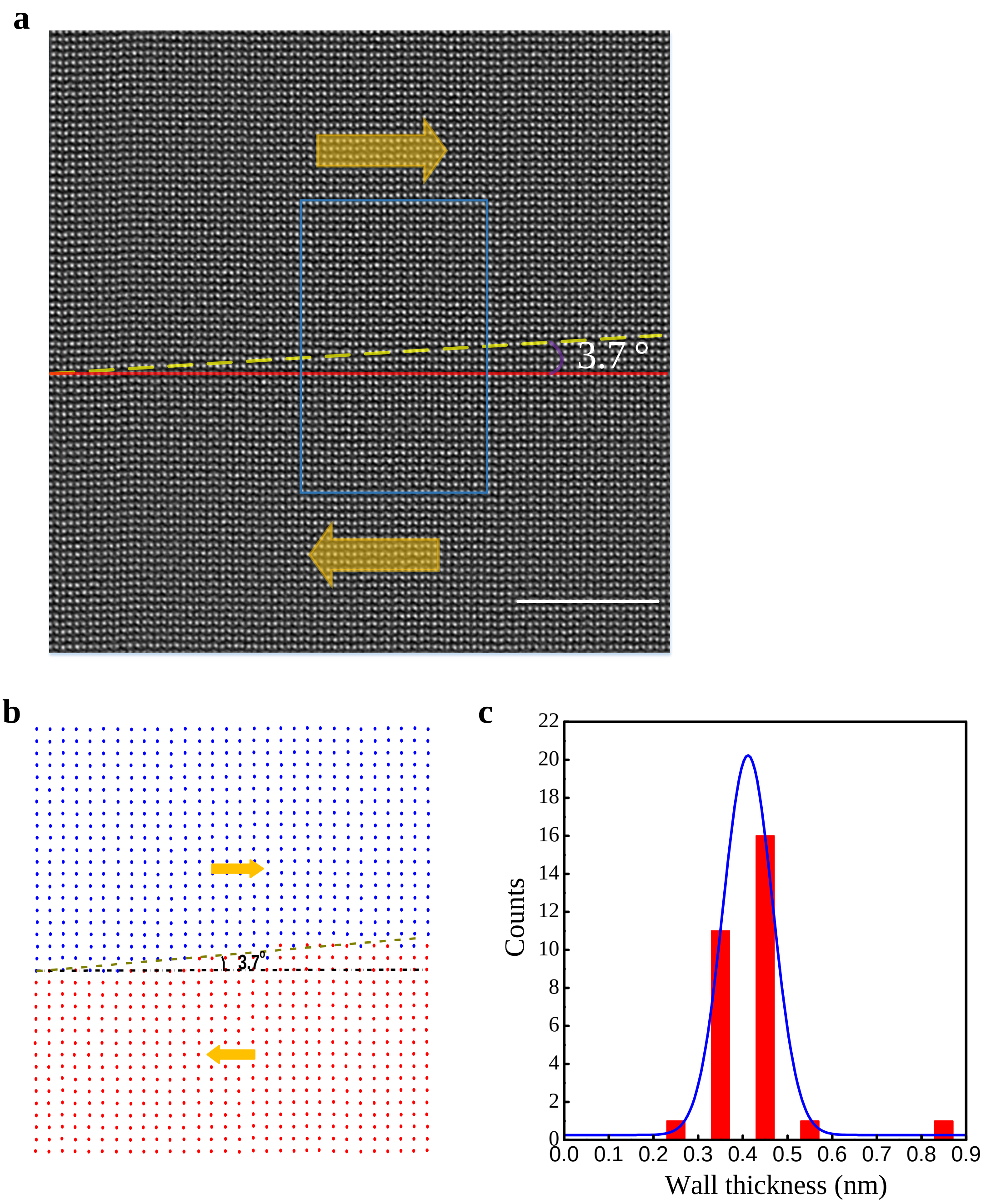

Supplementary Figure 11. Wall thickness for the D-G domain. **a** High-resolution LAADF-STEM cross-sectional image for S11 viewed along the $[\bar{1}100]$ direction in Fig. 5a in the main text, where the dashed line lineated the inclined wall. The scalar bar: 5 nm. **b** Red and blue dots correspond to the fitted Nb column positions for the framed region in panel a with opposite ionic displacements (thick arrow directions) across the wall region. **c** The statistical Gaussian distribution of wall thicknesses fitted by a solid line.

Supplementary References

1. Landauer, R. Electrostatic considerations in $BaTiO_3$ domain formation during polarization reversal. *J. Appl. Phys*. **28**, 227-234 (1957).
2. Turner, P. W. *et al*. Large carrier mobilities in $ErMnO_3$ conducting domain walls revealed by quantitative Hall-effect measurements. *Nano Lett*. **18**, 6381−6386 (2018).
3. Campbell, M. P. *et al.* Hall effect in charged conducting ferroelectric domain walls. *Nat. Commun*. **7**, 13764 (2016).
4. Jiang, J. *et al*. Temporary formation of highly conducting domain walls for non-destructive read-out of ferroelectric domain-wall resistance switching memories. *Nat. Mater.* **17**, 49−56 (2017).
5. Molotskii, M. *et al*. Ferroelectric domain breakdown. *Phys. Rev. Lett*. **90**, 107601 (2003).
6. Merz, W. J. Domain formation and domain wall motions in ferroelectric $BaTiO_3$ single crystals. *Phys. Rev*. **95**, 690–698 (1954).
7. Ma, J. *et al.* Controllable conductive readout in self-assembled, topologically confined ferroelectric domain walls. *Nat. Nanotech*. **13**, 947-952 (2018).
8. Volk, T. R., Gainutdinov, R. V. & Zhang, H. H. Domain-wall conduction in AFM-written domain patterns in ion-sliced $LiNbO_3$ films. *Appl. Phys. Lett.* **110**, 132905 (2017).
9. Lu, H. *et al*. Electrical tunability of domain wall conductivity in $LiNbO_3$ thin films. *Adv. Mater*. **48**, 1902890 (2019).
10. Han, H., Cai, L., Xiang, B., Jiang Y. & Hu, H. Lithium-rich vapor transport equilibration in single-crystal lithium niobate thin film at low temperature. *Opt. Mater. Express* **5**, 2634-2641 (2015).

11. Gonnissen, J. *et al*. Direct observation of ferroelectric domain walls in $LiNbO_3$: wall-Meanders, kinks, and local electric charges. *Adv. Funct. Mater*. **26**, 7599–7604 (2016).

12. den Dekker, A. J., Gonnissen, J., De Backer, A., Sijbers, J. & Van Aert, S. Estimation of unknown structure parameters from high-resolution (S)TEM images: What are the limits? *Ultramicroscopy* **134**, 34-43 (2013).

13. Salje, E. K. H. Ferroelastic materials. *Annu. Rev. Mater. Res.* **42**, 265-283 (2012).
